# Socioeconomic position and suicidal behaviour in rural Sri Lanka: a prospective cohort study of 168,000+ people

**DOI:** 10.1007/s00127-019-01672-3

**Published:** 2019-02-21

**Authors:** D. W. Knipe, D. Gunnell, R. Pieris, C. Priyadarshana, M. Weerasinghe, M. Pearson, S. Jayamanne, K. Hawton, F. Konradsen, M. Eddleston, C. Metcalfe

**Affiliations:** 1 Population Health Sciences, Bristol Medical School, University of Bristol, Canynge Hall 2.12, 39 Whatley Road, Bristol, BS8 2PS UK; 2 South Asian Clinical Toxicology Research Collaboration (SACTRC), Faculty of Medicine, University of Peradeniya, Peradeniya, Sri Lanka; 3 Pharmacology, Toxicology and Therapeutics, University/BHF Centre for Cardiovascular Science, University of Edinburgh, Edinburgh, UK; 4 Faculty of Medicine, University of Kelaniya, Kelaniya, Sri Lanka; 5 Department of Community Medicine, Faculty of Medicine and Allied Sciences, Rajarata University of Sri Lanka, Saliyapura, Anuradhapura, Sri Lanka; 6 Centre for Suicide Research, Department of Psychiatry, University of Oxford, Oxford, UK; 7 Department of Public Health, Faculty of Health and Medical Sciences, University of Copenhagen, Copenhagen, Denmark

**Keywords:** Suicide, Sri Lanka, Cohort study, Socioeconomic position, Low and middle income country

## Abstract

**Purpose:**

Lower socioeconomic position (SEP) is associated with an increased risk of suicidal behaviour in high income countries, but this association is not established in low- and middle-income countries (LMIC).

**Methods:**

We investigated the association of SEP with suicidal behaviour in a prospective cohort study of 168,771 Sri Lankans followed up for episodes of attempted suicide and suicide. SEP data were collected at baseline at the household and individual level at the start of the follow-up period. We used multilevel Poisson regression models to investigate the association of SEP at community, household and individual levels with attempted suicide/suicide.

**Results:**

Lower levels of asset ownership [IRR (95% CI) suicide 1.74 (0.92, 3.28); attempted suicide 1.67 (1.40, 2.00)] and education [suicide 3.16 (1.06, 9.45); attempted suicide 2.51 (1.70, 3.72)] were associated with an increased risk of suicidal behaviour. The association of these measures of SEP and attempted suicide was stronger in men than women. Individuals living in deprived areas [1.42 (1.16, 1.73)] and in households with a young female head of household [1.41 (1.04, 1.93)] or a temporary foreign migrant [1.47 (1.28, 1.68)] had an elevated risk of attempted suicide. Farmers and daily wage labourers had nearly a doubling in risk of attempted suicide compared to other occupations.

**Conclusions:**

Improved employment opportunities, welfare and mental health support services, as well as problem-solving skills development, may help support individuals with poorer education, farmers, daily wage labourers, individuals in young female-headed households and temporary foreign migrant households.

**Electronic supplementary material:**

The online version of this article (10.1007/s00127-019-01672-3) contains supplementary material, which is available to authorized users.

## Introduction

Suicide is a major cause of premature mortality worldwide, with nearly 80% of deaths occurring in low and middle income countries (LMIC) [1]. Over 50% of these suicide deaths in LMICs occur in the WHO’s South-East Asian region. Despite this, our knowledge of the factors that contribute to suicidal behaviour in these contexts is limited, partly due to the lack of available data.

Understanding the risk factors for suicidal behaviour is important to inform effective preventative strategies. The importance of suicide prevention has been globally recognised and the reduction of the suicide rate in countries has been included both in the World Health Organisation Global Mental Health Action Plan (2013) and as an indicator for the United Nations Sustainable Development Goals (Goal 3). There is a recognition that social factors, such as unemployment and socioeconomic position (SEP), play an important role in determining suicidal behaviour in high income countries [2, 3]. However, only two previous high quality prospective studies in LMIC in South and South-East Asia have explored this [4, 5] and these were limited by only exploring suicide as an outcome [4, 5], or restriction to a specific population (older adults [4, 5]). It has been suggested that greater levels of economic adversity lead to higher levels of anxiety, hopelessness and entrapment [6–8] and, therefore, can increase suicidal behaviour. Strategies designed to alleviate some of the associated distress in individuals of lower SEP may reduce the number of suicide attempts. In a cross-sectional investigation of the association between SEP and suicide attempts in Sri Lanka, we found that lower SEP was associated with an increased risk of attempted suicide in the last year [9]. A major limitation of our own study and of previous investigations [10] is the cross-sectional or retrospective nature of the study designs, which means that the temporal relationship between the exposure of interest and outcome is unclear. Our previous investigation also used a respondent-reported outcome (suicide attempts). This is a major limitation as self-report data can be unreliable [11] and it is possible that our results might have been affected by socially desirable responding bias.

We present the findings of a large prospective cohort study in rural Sri Lanka which aimed to answer the following questions: (1) is socioeconomic position associated with suicidal behaviour?; (2) is there evidence that any associations differ in males vs. females or young vs. old participants?; and (3) are there household or community level factors which contribute to the risk of suicide/attempted suicide?

## Methods

### Setting

Sri Lanka is a lower middle-income country situated off the south-east coast of India with a population of 20 million (Census 2011). Nearly, 80% of the population live in rural areas and roughly a third are employed in agriculture. The data used in this study were collected as part of the baseline survey of a large community-based randomised controlled trial in the Anuradhapura district, North Central province of Sri Lanka [12]. This is primarily an agricultural area.

### Participants

The cohort was based on participants aged 10+ years who took part in a large cluster randomised controlled trial (RCT) in rural Sri Lanka (i.e. a sub-sample of the main trial dataset) [12]. We assumed that children younger than 10 years would not exhibit suicidal behaviour. The methods of data collection have been previously described [12, 13] and are provided here in brief. Between December 2010 and February 2013, a door-to-door household survey was carried out by a team of high school graduates. All individuals living in the study area were eligible for inclusion. Details of the questionnaire have been previously published [9]. A face-to-face interview was conducted with an adult (≥ 18 years) household member after verbal consent was obtained. The respondent(s) provided consent and data for members of their household. For logistical purposes, the study area was split into 10 regions/bands. We only included data collected from bands 2–10, as data in band 1 for one of our main measures of SEP (household construction) were collected using slightly different definitions.

We assessed the representativeness of the study sample by comparing it to the population data collected in the 2011 census for the same region and found a similar age and sex pattern between the two datasets.

### Data structure

The data collected are clustered in nature as individuals live in households within communities. For the purpose of this analysis, we have used the same community boundaries which were used as part of the larger trial.

### Baseline survey

Data were collected on the characteristics of the household as well as on its members; these included measures of SEP. We also asked about whether someone in the household had previously attempted suicide. This was used to exclude individuals who had previously attempted suicide from the current study of incidence.

### Measures of SEP

The measures of SEP used in this analysis were either directly measured or derived. The measures were either at the individual, household or community level.

Individual measures of SEP included education and occupation. For each household member, the respondent was asked to report on the completed level of education and qualification received. For younger participants, the current education level was recorded. There were relatively few individuals with the highest level of qualification (university) and we, therefore, combined this category with those with an A-level qualification. Individuals were classed as having a salaried occupation if they received a monthly salary from a company and were not government employees, overseas workers or in the security forces, as these were separate categories in this data set. Examples of salaried employees were shop workers, insurance salespersons and bank employees.

A composite household measure of SEP [asset-based] was created combining data on household construction and motorised vehicle ownership. Asset-based measures show the most consistent associations with suicidal behaviour in LMIC [10]. We created a single asset score by dichotomising household construction (poor vs. moderate/high quality construction), and motorised vehicle ownership (ownership vs. non-ownership), and then combining these into a single score (see [9] for more details). In addition, we identified households with a young (≤ 40 years) female head of household, as these households are thought to be both materially and socially disadvantaged [14] and, in previous investigations, have shown to be at a slight elevated risk of attempted suicide [9]. We also included this as an individual level risk factor, to investigate any risk associated with being a young female head of household. At the individual level, the variable is possibly a marker of being widowed/divorced/separated and the financial/cultural stresses associated with overseeing a household. It may also be a marker of social isolation for the female. At a household level, this may be a marker of a lack of a father or authority figure, as well as economic difficulties. In addition, we identified households with a current temporary non-graduate foreign migrant [referred herein to as temporary foreign migrant households], as this has been linked previously to suicidal behaviour [15]. A temporary foreign migrant is someone who has emigrated for work abroad.

We derived a community level SEP measure based on the percentage of households with a poor asset score for each community and assigned this percentage to everyone in that community. We categorised individuals into five equal sized groups (quintiles).

### Other measures

The other individual measures used in this analysis include: (1) age (categorised into 4 groups: 10–25; 26–40; 41–55; and 56+ years of age. These age groups were based on the age-specific incidence of suicide attempts within the dataset); and (2) sex. We also explored the effect of controlling for a number of additional factors (supplementary material), but these were not included in the primary analyses as the association of these factors with the exposure and outcome is unclear.

### Outcome

In the randomised controlled trial forming the basis of the cohort study forming the basis of this paper, data were collected on all incidences of suicide and attempted suicide occurring in the study area and presenting at hospital (further details [12]) over a 3–5-year period. Data were collected from 11 small peripheral hospitals and 2 larger referral hospitals where sicker patients are cared for. Patients may first present at a peripheral hospital and then be transferred to one of the referral hospitals. These transfers were linked in the database. Suicide deaths that occurred before hospital presentation were identified through systematic surveys of police stations and coroners. Only suicide attempt/death cases resident in a village within the study boundary of the RCT were included in the analysis. Of the identified suicide/suicide attempts, 82% were linked back to an individual in the baseline survey and included in the analysis. We only have the age and sex of those cases not matched back to the baseline survey. We also revisited roughly 25% of households (*n* = 13,999 households) 3 years after the original baseline survey to ask about suicide attempts and deaths that occurred since the baseline survey that may have been missed by our surveillance system.

### Statistical analysis

We fitted mixed effects (multilevel) Poisson regression accounting for clustering at the household and community level. Mixed effects models account for the overdispersion in the data and the unit of analysis was individuals. We used total available follow-up calendar time as the offset, having accounted for the variable amount of time individuals spent in the residential address in the study area.

Two separate analyses were conducted, one with suicide deaths as the outcome and the other with suicide attempts. The analysis was done in several stages and included a primary and secondary set of analyses. For the primary analysis, we first ran models to estimate the age, sex, and intervention arm adjusted incidence rate ratios (IRR) for each of the six SEP measures (i.e. education; occupation; household asset score; young female headed household; household with a temporary foreign migrant; community level SEP) separately (6 models—see “Measures of SEP” for exposures). The intervention of the trial found no evidence of an effect and, therefore, we included both arms of the trial to maximise power, but we took the conservative approach of adjusting for the intervention arm to ensure that any differences we observed were not driven by the intervention.

Second, we investigated subgroup differences (sex and age), testing the null hypothesis of equal true associations between SEP and attempted suicide between men and women, and between the different age groups. We did this by fitting the above models with and without an interaction parameter (age was treated as a continuous variable), and then tested which of the two models was a better fit for the data using the likelihood ratio test. We present the *p* value for interaction and the results stratified by sex and age group.

Lastly, to investigate whether community and household factors (i.e. contextual factors) increased the risk of suicidal behaviour over and above that due to the characteristics of the people in these households/communities (i.e. compositional factors), we fitted three models which simultaneously modelled similar characteristics at each level to determine any effect of contextual factors. There were few suicide deaths during the follow-up period (*n* = 129) and, therefore, we restricted our analysis of suicides to the first and second models outlined. There were 87 (5%) individuals with more than one suicide attempt during the follow-up period. The earliest attempt was retained in the dataset and the individual’s follow-up censored at that time point (97 suicide attempts dropped).

For the secondary analysis, we explored the effect of adding in all the other potential confounders/mediators (supplementary material). We also fitted a single model with all exposures and additional factors from the previous model. All analyses were performed using Stata 15 (StataCorp, College Station, TX, USA).

### Missing data

A complete case analysis was conducted. The amount of missing data was very small for those cases who could be matched to their information in the baseline survey (0·5%).

### Ethics

Ethical approval was received from the research ethics committees of the University of Peradeniya and Rajarata University of Sri Lanka. This trial was registered with ClinicalTrials.gov, number NCT1146496.

## Results

### Population characteristics

**Fig. 1:**
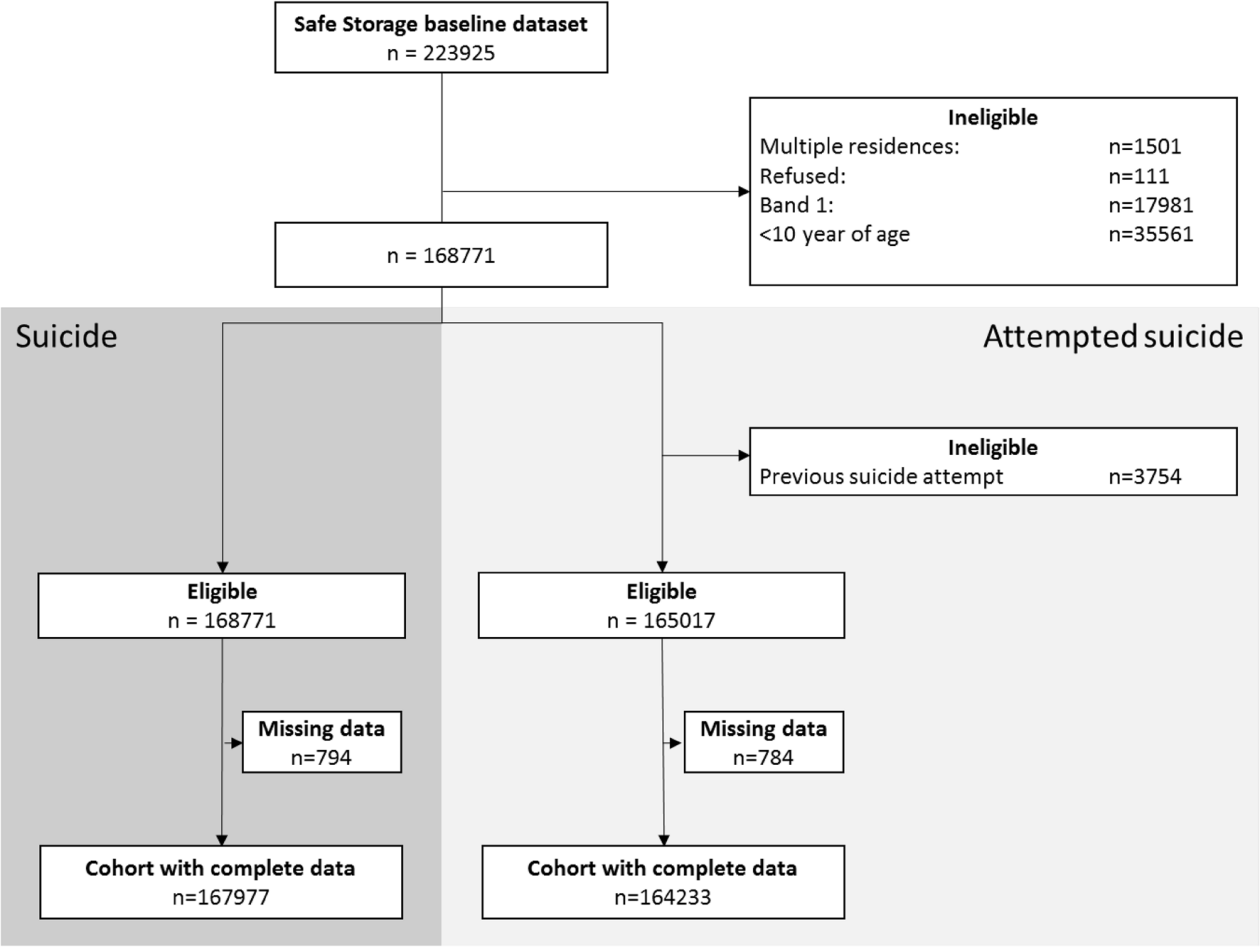
Flowchart of numbers of individuals included in the cohort analysis

There were 167,977 and 164,233 individuals for the suicide and attempted suicide outcome analysis respectively. (Fig. 1). Individuals with missing data tended to be older and have lower levels of education, though it is important to remember that a relatively small number of individuals had missing data. Table 1 presents the population characteristics of those included in the suicide attempt analysis (*n* = 164,233).

**Table 1:** Cohort characteristics

	Total population^a^*n* (%)	Outcome *n* (per 100,000 person years)	
		Suicide attempt	Suicide
*N*	164,233 (100)	1814 (340)	129 (23.5)
Community measured			
Deprivation^b^			
0–4.4%	32,299 (19.7)	274 (274.5)	25 (24.5)
4.5–5.2%	33,074 (20.1)	428 (383.0)	26 (22.6)
5.3–7.3%	33,127 (20.2)	297 (287.5)	26 (24.6)
7.4–9.4%	32,748 (19.9)	360 (331.7)	19 (17.0)
9.5–28.2%	32,985 (20.1)	455 (412.7)	33 (29.0)
Household measures			
Asset score			
High	107,289 (65.3)	1006 (287.2)	70 (19.6)
Middle	47,689 (29.0)	658 (429.5)	48 (30.2)
Low	9255 (5.6)	150 (496.9)	11 (34.5)
Non-graduate foreign employed	16,752 (10.2)	257 (590.1)	16 (35.5)
Young female headed household (≤ 40 years)	2407 (1.5)	44 (599.0)	1 (12.9)
Individual measures			
Sex			
Female	83,480 (50.8)	963 (338.8)	27 (9.2)
Male	80,753 (49.2)	851 (341.3)	102 (39.8)
Age group (years)			
10–25	50,461 (30.7)	1150 (716)	30 (18.2)
26–40	51,461 (31.3)	438 (279.2)	37 (22.8)
41–55	36,600 (22.3)	170 (135.0)	38 (29.4)
> 55	25,711 (15.7)	56 (62.1)	24 (26.3)
Education			
University/A-level	30,305 (18.5)	200 (217.0)	10 (10.7)
O-level	104,654 (63.7)	1421 (418.2)	81 (23.2)
Primary	24,194 (14.7)	162 (192.2)	33 (37.8)
Not attended	5080 (3.1)	31 (178.8)	5 (27.8)
Young female head of household (≤ 40 years)	784 (0.5)	4 (153.9)	0 (0)
Individual occupation			
Government worker/graduate foreign employed	6317 (3.9)	16 (80.9)	0 (0)
Farmer	28,280 (17.2)	252 (238.5)	43 (39.4)
Security forces	8498 (5.2)	49 (371.9)	7 (52.3)
Businessmen	3390 (2.1)	24 (184.9)	5 (37.7)
Self-employed	11,024 (6.7)	95 (260.5)	8 (21.2)
Non-graduate Foreign employed	4382 (2.7)	40 (1304.4)	1 (31.6)
Salaried employee	13,946 (8.5)	148 (425.3)	14 (39.1)
Daily wage labourer	9034 (5.5)	141 (478.7)	17 (54.3)
Unemployed/retired	18,225 (11.1)	165 (272.4)	12 (19.4)
House-worker/other	35,101 (21.4)	285 (230.8)	12 (9.4)
Student	26,036 (15.9)	599 (636.0)	10 (10.5)

^a^Characteristics of those included in the suicide attempt analysis

^b^% of households within a community with a low asset score categorised into quintiles

### Suicide

**Fig. 2:**
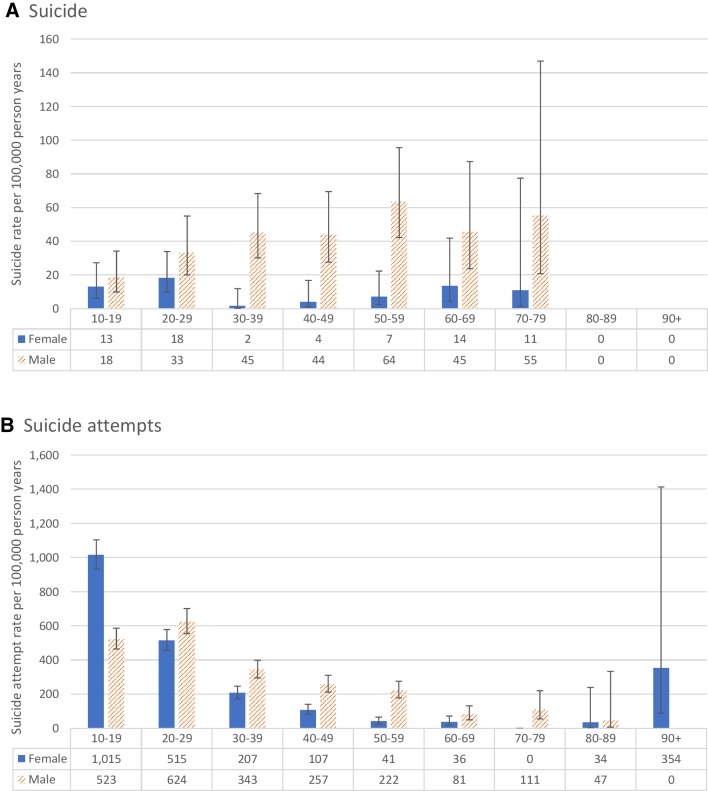
Annual incidence of **a** suicide and **b** suicide attempts in the study cohort by 10-year age group and sex

There were 129 suicide deaths (102 male and 27 female) included in the analysis, with an annual suicide rate of 23.5 per 100,000 (male 39.8 per 100,000, and female 9.2 per 100,000—M:F ratio 4.3:1, Table 1). The highest suicide rate was observed in the 50–59-year age group in males, and 20–29 in females (Fig. 2). Pesticide poisoning was the most common method of suicide (53%), followed by hanging (27%).

The analysis indicated that lower levels of asset ownership and lower levels of education were associated with a higher risk of suicide (Table 2). We were only able to test for interaction with sex and age for asset ownership, temporary foreign migrant households, and education; none of the tests showed evidence that sex or age altered any associations observed (*p* values for interaction ranged from 0.12 to 0.98).

**Table 2:** Risk of suicide by socioeconomic indicators stratified by age and sex

	IRR^a^ (95% CI)	Sex stratified IRR^b^ (95% CI)		Age stratified IRR^c^ (95% CI)				*p* value for interaction	
		Male	Female	10–25	26–40	41–55	56+	Sex	Age
Community measures									
Deprivation^d^									
0–4.4%	1	1	1	1	1	1	1	0.91	0.98
4.5–5.2%	0.96 (0.55, 1.66)	0.94 (0.51, 1.72)	1.15 (0.29, 4.58)	0.52 (0.12, 2.18)	1.75 (0.65, 4.75)	1.29 (0.49, 3.40)	0.28 (0.06, 1.35)		
5.3–7.3%	1.06 (0.60, 1.85)	0.95 (0.50, 1.78)	1.61 (0.44, 5.87)	1.15 (0.34, 3.86)	1.24 (0.41, 3.75)	0.86 (0.28, 2.61)	1.04 (0.35, 3.05)		
7.4–9.4%	0.72 (0.39, 1.31)	0.68 (0.35, 1.33)	0.91 (0.22, 3.80)	0.89 (0.26, 3.09)	0.80 (0.25, 2.64)	0.91 (0.32, 2.61)	0.29 (0.06, 1.41)		
9.5–28.2%	1.23 (0.73, 2.09)	1.18 (0.66, 2.12)	1.51 (0.42, 5.49)	1.83 (0.63, 5.36)	1.29 (0.44, 3.77)	1.04 (0.37, 2.92)	0.90 (0.30, 2.73)		
Household measures									
Asset score									
High	1	1	1	1	1	1	1	0.12	0.84
Middle	1.54 (1.07, 2.23)	1.65 (1.10, 2.47)	1.18 (0.49, 2.82)	1.14 (0.51, 2.56)	3.03 (1.57, 5.82)	0.92 (0.44, 1.93)	1.53 (0.66, 3.54)		
Low	1.74 (0.92, 3.28)	1.20 (0.52, 2.79)	3.69 (1.32, 10.32)	2.36 (0.79, 7.01)	0.85 (0.11, 6.4)	1.71 (0.59, 4.92)	1.58 (0.35, 7.08)		
Non-graduate foreign employed	1.28 (0.76, 2.17)	1.24 (0.68, 2.27)	1.45 (0.50, 4.21)	0.97 (0.29, 3.20)	1.03 (0.36, 2.91)	1.88 (0.79, 4.5)	1.33 (0.40, 4.46)	0.81	0.61
Young female headed household (≤ 40 years)	0.68 (0.10, 4.90)	–	–	–	–	–	–	–	–
Individual measures									
Education									
University/A-level	1	1	1	1	1	1	1	0.54	0.84
O-level	2.12 (1.10, 4.09)	2.71 (1.17, 6.26)	1.36 (0.45, 4.07)	1.69 (0.58, 4.89)	2.85 (0.87, 9.38)	4.26 (0.57, 31.88)	1.03 (0.23, 4.63)		
Primary	3.39 (1.63, 7.07)	4.24 (1.72, 10.48)	2.37 (0.56, 10.01)	3.61 (0.81, 16.19)	4.15 (0.93, 18.56)	8.87 (1.18, 66.42)	0.94 (0.20, 4.42)		
Not attended	3.16 (1.06, 9.45)	3.52 (0.87, 14.27)	3.53 (0.55, 22.71)	–	11.60 (1.94, 69.51)	3.67 (0.23, 58.72)	1.36 (0.19, 9.95)		
Individual occupation									
Government worker/graduate foreign employed	–	–	–	–	–	–	–	–	–
Farmer	1	–	–	–	–	–	–		
Security forces	0.53 (0.23, 1.22)	–	–	–	–	–	–		
Businessmen	1.04 (0.41, 2.62)	–	–	–	–	–	–		
Self-employed	0.55 (0.26, 1.18)	–	–	–	–	–	–		
Non-graduate foreign employed	0.26 (0.04, 1.93)	–	–	–	–	–	–		
Salaried employee	0.86 (0.45, 1.63)	–	–	–	–	–	–		
Daily wage labourer	1.24 (0.70, 2.19)	–	–	–	–	–	–		
Unemployed/retired	0.59 (0.30, 1.17)	–	–	–	–	–	–		
House-worker/other	0.65 (0.29, 1.42)	–	–	–	–	–	–		
Student	0.37 (0.16, 0.86)	–	–	–	–	–	–		

^a^Adjusted for age, sex and intervention arm

^b^Adjusted for age group and intervention arm

^c^Adjusted for sex and intervention arm

^d^% of households with a low asset score categorised into quintiles

### Suicide attempt

1814 individuals presented to the hospital following a suicide attempt during the follow-up period of the study, with an annual suicide attempt rate of 340 per 100,000 (male 341 per 100,000, and female 339 per 100,000—M:F ratio: 1:1, Table 1). The epidemiology of suicide attempt by age and sex differs to that of suicide, with the highest rate of attempts being observed in the 10–19-year age group in females, and 20–29-year group in males (Fig. 2).

Living in more deprived communities, lower levels of asset ownership and poorer education were associated with an increased risk of attempted suicide (Table 3). Living in a household with a current temporary foreign migrant was associated with a 47% increased risk of attempted suicide in household members, a similar elevated risk (41%) was observed in individuals living in a household with a young female head of household (Table 3). The risk of attempted suicide was not higher in young female heads of household compared to other individuals in this cohort. The attempted suicide rate in young female heads of household [153.9 per 100,000 (95% CI 57.8–410.0)] was consistent with rates observed in young male [379.0 (322.6–455.2)], older female [63.4 (38.9–103.5)], and older male [166 (142.0–195.1)] heads of household. Farmers appeared to have the highest risk of attempted suicide compared to almost all other occupations, except for daily wage labourers. In a post hoc analysis, we collapsed the other occupations which indicated a lower risk of attempted suicide and compared these individuals to farmers and daily wage labourers. This indicated that farmers had a 1.70 (1.46, 1.98) increased risk of attempted suicide (age and sex adjusted), with daily wage labourers experiencing an even higher risk [2.11 (1.76, 2.54)].

**Table 3:** Risk of attempted suicide by socioeconomic indicators stratified by age and sex

	Age + sex adjusted IRR (95% CI)^a^	Sex stratified IRR (95% CI)		Age stratified IRR (95% CI)^c^				*p* value for interaction	
		Male^b^	Female^b^	10–25	26–40	41–55	56+	Sex	Age
Community measured									
Deprivation^d^									
0–4.4%	1	1	1	1	1	1	1	0.37	0.04
4.5–5.2%	1.31 (1.06, 1.62)	1.13 (0.85, 1.51)	1.52 (1.18, 1.96)	1.37 (1.08, 1.74)	1.40 (0.97, 2.02)	0.94 (0.56, 1.57)	1.40 (0.59, 3.32)		
5.3–7.3%	1.05 (0.85, 1.30)	0.92 (0.69, 1.23)	1.18 (0.91, 1.53)	1.09 (0.85, 1.39)	1.19 (0.82, 1.73)	0.65 (0.36, 1.17)	0.49 (0.17, 1.43)		
7.4–9.4%	1.19 (0.97, 1.46)	1.07 (0.81, 1.41)	1.31 (1.01, 1.69)	1.21 (0.96, 1.54)	1.31 (0.91, 1.88)	0.94 (0.56, 1.58)	0.89 (0.35, 2.25)		
9.5–28.2%	1.42 (1.16, 1.73)	1.40 (1.07, 1.83)	1.43 (1.12, 1.83)	1.33 (1.06, 1.68)	1.56 (1.10, 2.22)	1.81 (1.14, 2.87)	1.11 (0.46, 2.68)		
Household measures									
Asset score									
High	1	1	1	1	1	1	1		
Middle	1.47 (1.33, 1.63)	1.75 (1.51, 2.02)	1.27 (1.1, 1.46)	1.39 (1.22, 1.58)	1.77 (1.44, 2.16)	1.63 (1.18, 2.25)	1.16 (0.67, 2.01)	0.01	0.7
Low	1.67 (1.40, 2.00)	1.71 (1.32, 2.22)	1.65 (1.3, 2.09)	1.61 (1.29, 2.01)	2.42 (1.71, 3.43)	1.26 (0.66, 2.39)	0.57 (0.14, 2.38)		
Non-graduate foreign employed	1.47 (1.28, 1.68)	1.52 (1.25, 1.86)	1.42 (1.18, 1.71)	1.43 (1.2, 1.71)	1.47 (1.13, 1.93)	1.84 (1.20, 2.82)	1.59 (0.75, 3.37)	0.62	0.96
Young female headed household (≤ 40 years)	1.41 (1.04, 1.93)	1.73 (1.09, 2.76)	1.27 (0.85, 1.91)	1.70 (1.19, 2.44)	1.13 (0.60, 2.13)	–	–	0.53	0.05
Individual measures									
Education									
University/A-level	1	1	1	1	1	1	1	< 0.001	< 0.001
O-level	2.17 (1.87, 2.52)	3.31 (2.48, 4.40)	1.88 (1.58, 2.25)	2.03 (1.70, 2.41)	2.99 (2.14, 4.17)	2.59 (1.25, 5.36)	–		
Primary	2.25 (1.81, 2.80)	4.37 (3.11, 6.13)	1.28 (0.91, 1.81)	1.05 (0.71, 1.55)	4.29 (2.77, 6.64)	3.93 (1.87, 8.27)	–		
Not attended	2.51 (1.70, 3.72)	5.83 (3.44, 9.88)	1.68 (0.89, 3.17)	0.88 (0.28, 2.78)	3.60 (1.59, 8.13)	6.70 (2.8, 16.00)	–		
Young female head of household (≤ 40 years)	0.50 (0.19, 1.34)	–	–	–	–	–	–	–	–
Individual occupation^a^									
Government worker/graduate foreign employed	0.25 (0.15, 0.42)	0.33 (0.19, 0.60)	0.22 (0.08, 0.62)	0.12 (0.03, 0.50)	0.23 (0.11, 0.50)	0.49 (0.22, 1.07)	–	< 0.001	< 0.001
Farmer	1	1	1	1	1	1	1		
Security forces	0.34 (0.25, 0.46)	0.32 (0.23, 0.44)	0.57 (0.14, 2.40)	0.34 (0.22, 0.53)	0.27 (0.16, 0.47)	0.50 (0.18, 1.38)	–		
Businessmen	0.66 (0.43, 1.01)	0.64 (0.39, 1.04)	0.91 (0.38, 2.18)	0.69 (0.31, 1.54)	0.45 (0.22, 0.94)	0.98 (0.44, 2.17)	1.36 (0.31, 5.84)		
Self-employed	0.79 (0.62, 1.01)	0.82 (0.62, 1.07)	1.01 (0.58, 1.76)	0.73 (0.47, 1.15)	0.86 (0.61, 1.23)	0.61 (0.32, 1.17)	1.18 (0.44, 3.16)		
Non-graduate foreign employed	0.62 (0.44, 0.87)	0.40 (0.20, 0.81)	1.31 (0.80, 2.14)	0.45 (0.24, 0.82)	0.99 (0.63, 1.57)	0.46 (0.11, 1.92)	–		
Salaried employee	0.52 (0.42, 0.65)	0.46 (0.35, 0.61)	0.98 (0.63, 1.51)	0.44 (0.31, 0.61)	0.52 (0.35, 0.77)	0.67 (0.31, 1.48)	0.72 (0.10, 5.36)		
Daily wage labourer	1.26 (1.02, 1.56)	1.23 (0.98, 1.54)	1.76 (0.97, 3.22)	1.10 (0.75, 1.61)	1.49 (1.07, 2.07)	1.27 (0.79, 2.04)	1.43 (0.48, 4.19)		
Unemployed/retired	0.74 (0.59, 0.91)	1.07 (0.83, 1.38)	0.88 (0.57, 1.38)	0.61 (0.44, 0.86)	0.95 (0.56, 1.60)	0.79 (0.36, 1.74)	0.83 (0.43, 1.59)		
House-worker/other	0.60 (0.49, 0.73)	0.68 (0.30, 1.55)	1.12 (0.77, 1.63)	0.44 (0.31, 0.62)	1.05 (0.75, 1.47)	1.10 (0.62, 1.94)	0.70 (0.22, 2.29)		
Student	0.69 (0.57, 0.82)	0.45 (0.36, 0.58)	1.47 (0.99, 2.18)	0.56 (0.42, 0.75)	–	–	–		

^a^Adjusted for age, sex and intervention arm

^b^Adjusted for age group and intervention arm

^c^Adjusted for sex and intervention arm

^d^% of households with a low asset score categorised into quintiles

The association between educational level and the risk of attempted suicide was different for men and women, with the risk being about three times higher in males than females (*p* value for interactions < 0.001) (Table 3). There was also evidence that the association between occupation (*p* (interaction)  < 0.001), assets (*p* (interaction) = 0.01) and suicide attempt risk was different for males and females. Using the post hoc occupation groups (farmers, daily wage labourers and other occupations), there was only statistical evidence of an increased risk of attempted suicide in males [farmers 1.80 (1.51, 2.16); daily wage 2.28 (1.86, 2.79)] and not females [farmers 0.94 (0.65, 1.35); daily wage 1·63 (0·98, 2·71)] for both famers and daily wage labourers compared to other occupations. There was evidence that the strength of associations observed between attempted suicide with education (*p* (interaction) < 0.001) and occupation (*p* (interaction) < 0.001) differed between age groups. The risk was greater for those who did not attend school in the older age groups than the younger ones. There was also weaker statistical evidence (*p* (interaction)  =  0.05) to suggest that younger individuals in households with a female head of household were at a greater risk of attempted suicide.

Table 4 shows the findings of the three models investigating whether there was evidence of contextual factors contributing to the risk of attempted suicide in this cohort. The analysis suggests that living in deprived communities and in households with lower levels of assets increases the risk of attempted suicide independent of the SEP status (in this case education) of the individual living in that community/household (Model A). There was also evidence that living in a household with a non-graduate foreign employed individual increased the risk of attempted suicide independent of individual occupation status (Model B). Furthermore, there was an increased risk of attempted suicide (72%) in individuals with a young female head of household, independent of any associated risk to the young female head of household themselves (Model C). Our analysis suggests that being a young female head of household was associated with a reduced risk of attempted suicide.

**Table 4:** Assessment of whether risk of attempted suicide is influenced by contextual socioeconomic indicators over and above individual-level indicators—Model A: socioeconomic conditions (community, household and individual); Model B: non-graduate foreign employed (household and individual); Model C: young female head of household (household and individual)

	Suicide Attempt IRR (95% CI)^a^		
	Model A	Model B	Model C
Community measures			
Deprivation^b^			
0–4.4%	1	–	–
4.5–5.2%	1.26 (1.03, 1.55)	–	–
5.3–7.3%	1.00 (0.82, 1.23)	–	–
7.4–9.4%	1.11 (0.91, 1.36)	–	–
9.5–28.2%	1.26 (1.04, 1.54)	–	–
Household measures			
Asset score			
High	1	–	–
Middle	1.38 (1.25, 1.53)	–	–
Low	1.48 (1.24, 1.78)	–	–
Non-graduate foreign employed	–	1.58 (1.36, 1.83)	–
Young female headed household (≤ 40 years)	–	–	1.72 (1.24, 2.39)
Individual measures			
Education			
University/A-level	1	–	–
O-level	2.05 (1.76, 2.38)	–	–
Primary	2.03 (1.63, 2.53)	–	–
Not attended	2.18 (1.47, 3.22)	–	–
Young female head of household (≤ 40 years)	–	–	0.30 (0.11, 0.84)
Individual occupation			
Government worker/Graduate foreign employed	–	0.26 (0.15, 0.43)	–
Farmer	–	1	–
Security forces	–	0.34 (0.25, 0.47)	–
Businessmen	–	0.67 (0.44, 1.02)	–
Self-employed	–	0.79 (0.62, 1.01)	–
Non-graduate foreign employed	–	0.42 (0.29, 0.60)	–
Salaried employee	–	0.52 (0.42, 0.65)	–
Daily wage labourer	–	1.25 (1.01, 1.54)	–
Unemployed/retired	–	0.73 (0.59, 0.91)	–
House-worker/other	–	0.60 (0.49, 0.72)	–
Student	–	0.69 (0.57, 0.83)	–

^a^All models adjusted for age, sex and intervention arm, as well as those listed in the table

^b^% of households with a low asset score categorised into quintiles

In the secondary analysis, adjusting for the other potential confounders/mediators made little difference to the associations in the age and sex adjusted analysis (Supplementary table 1—Model 1). The simultaneous adjustment of all SEP measures with all other potential confounding/mediating factors resulted in a reduction of the risk observed with lower levels of education, assets and deprivation.

## Discussion

### Main findings

Indicators of low SEP were associated with an increased risk of suicide and attempted suicide. Specifically, lower levels of asset ownership and education were associated with an increased risk of both suicide and attempted suicide. The association of these measures of SEP and suicide attempt risk was considerably stronger in men than women. Individuals living in areas with higher levels of deprivation, and in households with a female head of household or a temporary foreign migrant had a moderately elevated risk of attempted suicide. In terms of occupation, we found that farmers and daily wage labourers had nearly a doubling in risk of attempted suicide.

### Comparison to other studies

Our comparison to existing literature is restricted to evidence from LMIC in Asia. Suicide is a significant public health issue in other LMIC regions (e.g. Western Pacific, Africa), but these settings are likely to differ contextually to the region we have conducted our research. Therefore, we have chosen to restrict our comparison to other Asian studies. The suicide rate observed in this study (23.5 per 100,000) is higher than Sri Lanka’s national suicide rate (16.9 per 100,000 in 2016), but similar to the rate observed in the Anuradhapura district (27.8 per 100,000 in 2016). Several studies have investigated the association between SEP and suicidal behaviour in LMIC [10], but there have only been two cohort studies (India and China) comparable to the current study (India—*n* = 131,728, 385 deaths [4]; China—*n* = 158,666, 197 deaths [5]). Both these studies only included suicide as an outcome (hospital/death records) and included only older adults (> 34 years); neither of the studies found statistical evidence of an association between lower levels of education and suicide. The study from India also found no evidence of an association between lower levels of asset and suicide risk [4].

There are no longitudinal studies investigating the association of SEP and attempted suicide in this region. The only evidence from this region comes from cross-sectional and case–control studies, which have shown a higher risk of suicide attempts in individuals with lower levels of assets/wealth, with an apparent dose response relationship [16, 17]. Our own cross-sectional investigation (on the same population) indicated a higher risk of self-reported attempted suicide with lower levels of asset ownership [9] (the same measure used in the present study). The findings of this prospective study, however, indicate a lower risk estimate with lower levels of asset ownership (low asset score—IRR 1.67 vs. OR 3.21). It is possible that our previous findings were affected by individuals from higher SEP backgrounds being less likely to report suicide attempts (social desirability bias). The use of self-report outcomes may thereby overestimate the risk in lower SEP groups.

Previous cohort studies have shown that individuals in manual occupations in this region were at a higher risk of suicidal behaviour (RR 1.2–1.4), which is consistent with evidence from this study [4, 5]. Findings from other LMICs in South and South-East Asia have shown inconsistent evidence in relation to the association with unemployment and suicidal behaviour, with the majority of studies reporting effect estimates which include the null (i.e. no association) [10]. The definition of unemployment varies between studies, and not all studies define unemployment making comparison and interpretation difficult. Previous studies have also tended to adjust for other measures of SEP, [5, 18–21] which is likely to be an over adjustment and may, therefore, explain why an association with unemployment and suicidal behaviour was not reported.

Living in a household with a young female head of household (≤ 40 years) was associated with increased risk of attempted suicide. Targeting prevention efforts in young female headed households may appear attractive as identifying this group of individuals would be relatively easy, but (assuming a causal relationship) less than 1% (population attributable risk proportion) of suicide attempts in the population are in individuals from these households. Prevention efforts for risk factors/exposures that are related to a higher proportion of the population attributable risk are likely to have a bigger impact.

In Sri Lanka, a large number of individuals migrate overseas for temporary work (2–3 years) in low skilled employment. Suicidal behaviour has been linked in previous qualitative work to individuals in these households [15, 22]. This study provides strong evidence that individuals in these households are vulnerable, but we estimate the population attributable risk for this factor is only 5%. Despite the strength of the longitudinal nature of this dataset, we were only able to look at the impact of current migration. We do not have data on the factors that were present prior to the migration which might have been important drivers of migration. In other words, the migration may be an intermediary factor between a pre-existing vulnerability and poor health outcomes.

### Implications

If causal the associations reported in this study are causal, they suggest that stable employment prospects need to be improved for rural Sri Lankans; this may include increasing opportunities for business establishment. In addition, other possible interventions to support these groups could include welfare support, skills development (e.g. improved budgeting and problem-solving skills), and mental health support.

### Strengths and limitations

This is the first large cohort study conducted in a general population sample (including all at risk ages) investigating the association of SEP and suicide and attempted suicide in an LMIC. The outcome measures used in this study were objectively measured and did not rely on self-report.

There are, however, important limitations to this study. First, the data included cases of suicide/attempted suicide collected in hospital or through coroners. We may have missed some suicide deaths and attempts. It may be that not all those who attempt suicide go to hospital but instead may seek alternative health care. In the re-survey of 25% households, we found only 8% of cases of self-harm did not present to hospital in the study area. However, it is possible that some suicide deaths may have occurred outside the study area and be missed in this analysis. Second, the analysis only included suicide deaths and suicide attempts which were linked back to individuals in the baseline survey. Eighteen percent of cases were not matched back to individuals. It is possible that by excluding these individuals we may have biased our findings; particularly if these individuals were more likely to be from a certain SEP background. We were unable to test for this, but we did observe that unmatched cases were more likely to be female and younger. Third, the contextual factors (especially at the community level) were derived from a household level factor and are not truly contextual in the way that infrastructure indicators are (e.g. number of school or health clinics). Lastly, the analysis only includes 129 suicide deaths. Given the small number of cases in the dataset, the study is likely to be underpowered to detect anything but large differences between SEP groups.

## Conclusion

This large prospective cohort study from rural Sri Lanka indicates that lower levels of SEP are associated with an increased risk of suicidal behaviour. Several vulnerable groups were identified in this study: those with lower levels of education, farmers, daily wage labourers, individuals in female-headed households and temporary foreign migrant households. Improving opportunities for stable employment and increased welfare support may help these vulnerable groups by assisting to alleviate distress associated with economic adversity.

## Electronic supplementary material

Below is the link to the electronic supplementary material.

Supplementary material 1 (DOCX 23 KB)
