# Supplementary material for: Socioeconomic position and suicidal behaviour in rural Sri Lanka: a prospective cohort study of 168,000+ people
Source: Soc Psychiatry Psychiatr Epidemiol. 2019 Feb 21;54(7):843–55. doi: 10.1007/s00127-019-01672-3 (PMC6656893; doi:10.1007/s00127-019-01672-3)
Supplement: Supplementary file 1 — Supplementary material 1 (DOCX 23 KB) [file 127_2019_1672_MOESM1_ESM.docx]

**Supplementary Material**

**Supplementary methods:**

We explored the effect of controlling a range of other potential confounding/mediating factors as a secondary analysis. These factors included: i) household pesticide access (defined as the household either storing or using pesticides); ii) absence of young children in the household (defined as households with children under the age of 10); iii) ‘problem’ alcohol use (household respondent(s) were asked whether someone in the household consumed alcohol, and then whether this alcohol consumption was perceived by anyone in the household as a ‘problem’); and iv) the number of generations in the household (we created an indirect measure of the number of generations in a household by splitting the occupants into four age categories: <10; 10-25, 26-65 and 65+. We then counted how many of these age categories there were in each household: 1, 2, 3 or 4). At a community level we also derived two additional measures based on the percentage of households that had: i) access to pesticides (as a measure of environmental exposure to pesticides which has previously been linked to suicidal ideation [26]); and ii) ‘problem’ alcohol use (a measure of community level alcohol misuse, which may increase suicidal behaviour through social fragmentation or increased community level violence [27]). Both these measures were categorised into quintiles.

**Supplementary results:**

**Table S1 - Risk of attempted suicide by socioeconomic indicators adjusting for potential confounders/mediators**

|  |  | Suicide Attempt IRR (95% CI) | |
| --- | --- | --- | --- |
|  |  | Model A | Model B |
| Community measures | |  |  |
| Deprivation* | |  |  |
|  | 0-4.4% | 1 | 1 |
|  | 4.5-5.2% | 1.28 (1.04, 1.56) | 1.21 (0.99, 1.47) |
|  | 5.3-7.3% | 1.02 (0.83, 1.26) | 0.97 (0.79, 1.19) |
|  | 7.4-9.4% | 1.18 (0.97, 1.45) | 1.09 (0.9, 1.33) |
|  | 9.5-28.2% | 1.43 (1.18, 1.74) | 1.26 (1.04, 1.52) |
| Household measures | |  |  |
| Asset score | |  |  |
|  | High | 1 | 1 |
|  | Middle | 1.48 (1.34, 1.64) | 1.34 (1.21, 1.49) |
|  | Low | 1.69 (1.41, 2.02) | 1.41 (1.17, 1.69) |
| Non-graduate foreign employed | | 1.47 (1.28, 1.68) | 1.52 (1.31, 1.76) |
| Young female headed household (≤40 years) | | 1.43 (1.05, 1.95) | 1.47 (1.06, 2.04) |
| Individual measures | |  |  |
| Education | |  |  |
|  | University/A-level | 1 | 1 |
|  | O-Level | 2.11 (1.81, 2.45) | 1.81 (1.55, 2.12) |
|  | Primary | 2.18 (1.75, 2.72) | 1.65 (1.32, 2.07) |
|  | Not attended | 2.47 (1.67, 3.66) | 1.78 (1.2, 2.65) |
| Young female head of household (≤40 years) | | 0.51 (0.19, 1.37) | 0.28 (0.1, 0.8) |
| Individual occupation | |  |  |
|  | Government worker/Graduate foreign employed | 0.26 (0.16, 0.43) | 0.41 (0.24, 0.69) |
|  | Farmer | 1 | 1 |
|  | Security forces | 0.34 (0.25, 0.46) | 0.38 (0.28, 0.52) |
|  | Businessmen | 0.68 (0.44, 1.04) | 0.81 (0.53, 1.23) |
|  | Self-employed | 0.79 (0.62, 1.01) | 0.83 (0.65, 1.06) |
|  | Non-graduate Foreign employed | 0.63 (0.45, 0.89) | 0.42 (0.29, 0.61) |
|  | Salaried employee | 0.53 (0.42, 0.66) | 0.56 (0.45, 0.69) |
|  | Daily Wage labourer | 1.26 (1.02, 1.57) | 1.14 (0.92, 1.42) |
|  | Unemployed/retired | 0.74 (0.6, 0.92) | 0.78 (0.63, 0.97) |
|  | House-worker/Other | 0.59 (0.49, 0.72) | 0.61 (0.5, 0.74) |
|  | Student | 0.68 (0.57, 0.82) | 0.72 (0.6, 0.87) |

* % of households with a low asset score categorised into quintiles

Model A – Adjusted for age, sex, intervention arm, pesticide access, absence of children in the household; ‘problem’ alcohol use; multi-generational households; community pesticide exposure; and community alcohol use

Model B – Adjusted for age, sex, intervention arm, pesticide access, absence of children in the household; ‘problem’ alcohol use; multi-generational households; community pesticide exposure; community alcohol use and all SEP measures listed in the table
